# Malignant Mesenchymal Tumors of the Breast: Current Challenges and New Perspectives on Primary Sarcomas and Malignant Phyllodes Tumors

**DOI:** 10.3390/life15040673

**Published:** 2025-04-20

**Authors:** Flavia De Lauretis, Alejandro Martin Sanchez, Cristina Accetta, Beatrice Carnassale, Sabatino D’Archi, Alba Di Leone, Antonio Franco, Federica Gagliardi, Stefano Magno, Elena Jane Mason, Francesca Moschella, Lorenzo Scardina, Marta Silenzi, Angela Bucaro, Chiara V. Pirrottina, Nicoletta D’Alessandris, Antonino Mulè, Angela Santoro, Fabio Marazzi, Valeria Masiello, Alessandra Fabi, Armando Orlandi, Antonella Palazzo, Ida Paris, Maria Pia Foschini, Riccardo Masetti, Gianluca Franceschini

**Affiliations:** 1 Multidisciplinary Breast Center, Dipartimento Scienze della Salute della Donna e del Bambino e di Sanità Pubblica, Fondazione Policlinico Universitario A. Gemelli IRCCS, 00168 Rome, Italy; 2 Breast Surgery, Center for Women’s and Newborn Health, Isola Tiberina Hospital, Gemelli Isola, 00153 Rome, Italy; 3 Unità di Ginecopatologia e Patologia Mammaria, Dipartimento Scienze della Salute della Donna e del Bambino e di Sanità Pubblica, Fondazione Policlinico Universitario A. Gemelli IRCCS, 00168 Rome, Italy; 4 Division of Radiotherapy, Dipartimento di Diagnostica per Immagini, Radioterapia Oncologica ed Ematologia, Fondazione Policlinico Universitario A. Gemelli IRCCS, 00168 Rome, Italy; 5 Division of Medical Oncology, Fondazione Policlinico Universitario A. Gemelli IRCCS, 00168 Rome, Italy; 6 Breast Unit, Bellaria Hospital, AUSL Bologna, Department of Biomedical and Neuromotor Sciences, University of Bologna, 40138 Bologna, Italy

**Keywords:** mesenchymal tumors, breast neoplasms, phyllodes tumors, breast sarcomas, therapeutic approaches, surgical treatment, chemotherapy, radiotherapy, prognosis, local recurrence

## Abstract

Mesenchymal tumors of the breast constitute a rare and heterogeneous group of neoplasms, representing only 0.5% to 1% of all breast tumors. Originating from mesenchymal tissues, these tumors include various histological subtypes. They are particularly aggressive, characterized by a high propensity for local recurrence and an overall poor prognosis. The rarity of these cases has impeded the development of comprehensive clinical studies, leading to a lack of standardized diagnostic protocols and treatment guidelines. This review provides a thorough synthesis of current knowledge on breast mesenchymal tumors with a specific focus on malignant variants such as phyllodes tumors and breast sarcomas. It also addresses the diagnostic challenges faced by clinicians, evaluates current therapeutic strategies, and emphasizes the crucial role of surgical treatment. Additionally, it examines the evolving roles of chemotherapy and radiotherapy in enhancing patient outcomes.

## 1. Introduction

Most breast cancers originate from epithelial tissue, but there is a heterogeneous group of non-epithelial tumors arising from mesenchymal tissues of the breast, which comprise 0.5 to 1% of all breast tumors [1].

According to the World Health Organization (WHO), mesenchymal tumors include 17 lesions of vascular, lipomatous, nerve sheath, myofibroblastic, and myogenic origin. They range from benign to highly malignant and very common to extremely rare [2].

Malignant mesenchymal tumors of the breast have a different natural history, treatment response, and prognosis as compared with epithelial ones. Due to their rarity, most publications consist of case reports or limited retrospective series with inconsistent inclusion criteria and excessively broad observational periods, combining cases from the early 20th century with those treated using the latest diagnostic and therapeutic protocols [3,4,5] (Table 1). Notably, malignant phyllodes tumors currently fall under breast cancer guidelines [6]. In contrast, breast sarcomas are considered within the broader category of soft tissue sarcomas, following the recommendations for sarcomas arising in other anatomical sites [7,8]. This leads to a heterogeneous interpretation and formulation of current guidelines regarding the clinical management of these rare tumors, particularly on the efficacy of complementary treatments [9].

This review provides an overview of current evidence about the most aggressive forms of mammary tumors, specifically malignant phyllodes tumors (MPTs) and primary breast sarcomas (PBSs). It focuses on the proven effectiveness of surgery, as well as the reported results of chemotherapy and radiotherapy.

## 2. Phyllodes Tumors

Phyllodes tumors (PTs) of the breast are neoplasms composed of both epithelial and stromal elements [4,5,6,7,8,9,10,11].

They are characterized by rapid dimensional growth and often present as fibroadenomas on initial radiological examinations (ultrasound and mammography); cytological examination via fine needle aspiration and histological examination via core biopsy may sometimes be inadequate to reliably distinguish PTs from fibroadenomas [12].

Excisional biopsy is recommended for diagnostic purposes in cases of rapid growth of fibroadenoma-like lesions, which exhibit variable biological behavior. Based on histopathological characteristics, PTs are categorized as benign (60–75%), borderline (15–20%), or malignant (10–20%), which may occasionally transform histologically into sarcomatous lesions [13,14].

National Comprehensive Cancer Network (NCCN) guidelines recommend radical excision of the lesion with ≥1 cm negative margins. Axillary lymph node metastases are rare; sentinel lymph node biopsy and/or axillary lymphadenectomy are, therefore, not indicated unless the lymph nodes are suspected at clinical staging [7].

### 2.1. Epidemiology and Risk Factors

PTs represent approximately 0.3–0.9% of all breast neoplasms. Among all PTs, malignant ones constitute 10–20% of cases, with an annual incidence of 2.1 cases per million women [15]. No etiological or risk factors have been described for the development of these neoplasms except for Li–Fraumeni syndrome, a rare autosomal dominant condition characterized by the development of multiple tumor types [16].

### 2.2. Diagnosis

PTs are a complex type of breast lesion that pose challenges for diagnosis. Ultrasound may show hypoechoic, heterogeneous, or complex cystic and solid echo patterns [17]. Features such as lobulated shape, heterogeneous internal echo pattern, and absence of microcalcification are significant sonographic features used to favor PTs over fibroadenomas [11,18].

Specific sonographic features, such as liquefaction and a heterogeneous inner echo, may indicate a higher risk of MPT of the breast [19].

On Magnetic Resonance Imaging (MRI), PTs appear as oval, well-circumscribed, and isointense on T1-weighted images, and heterogeneously hyperintense on T2-weighted images [18]. Certain MRI findings can aid in assessing the risk of malignancy. Features such as non-circumscribed margins, cystic components, irregular cyst walls, peritumoral edema, a low signal intensity on T2-weighted images, and a low apparent diffusion coefficient (ADC) are associated with a higher histologic grade in histopathology [17].

### 2.3. Pathology Assessment

The World Health Organization categorizes PTs as benign, borderline, or malignant based on factors such as stromal cellularity, stromal overgrowth, nuclear atypia, mitotic activity, tumor margins, and the presence of malignant heterologous elements [20].

Key diagnostic features of benign PTs include a cellular stroma with a subepithelial distribution, a low mitotic count of fewer than five mitoses per 10 high-power fields, and a varied stroma that can include hyalinized or myxoid areas. Borderline tumors, according to the WHO classification, have intermediate characteristics, exhibiting microscopic invasion at the tumor margins along with moderate cellularity and pleomorphism [2,21].

MPTs are characterized by significant nuclear pleomorphism, diffuse stromal cellularity, stromal overgrowth, mitotic activity of more than 10 mitoses per 10 HPFs, and infiltrative borders. Differentiating MPTs from pure or metastatic sarcomas can be quite challenging. There is a notable risk of distant metastasis—reportedly up to 16%—especially to the lungs and bones, while involvement of the axillary lymph nodes is rare.

Histological factors associated with distant metastases include tumor size over 7 cm, infiltrative borders, marked stromal overgrowth, high stromal cellularity, >5 mitoses per 10 HPFs, and necrosis. MPTs are often resistant to chemotherapy, and the prognosis for metastatic cases is poor [19,22].

Immunohistochemical analysis indicates that benign PTs display low positivity for p53, Ki-67, CD117, EGFR, p16, and VEGF, with an increasing positivity in MPTs.

Conversely, Lenhard and colleagues have defined stroma-rich PTs with a bland appearance as typical CD34–positive, while high-grade MPTs usually show loss or a lesser grade of CD34 staining [23].

Mutations in genes such as TP53, RB1, NF1, PIK3CA, and ERBB4 have been found in MPTs, suggesting potential therapeutic targets. Additionally, mutations in genes such as TP53, RB1, NF1, PIK3CA, and ERBB4 have been found in MPTs, suggesting potential therapeutic targets [11,24,25].

In conclusion, accurate histological assessment of PTs, along with immunohistochemical and genetic analyses, help differentiate between benign, borderline, and MPTs. This comprehensive approach guides appropriate clinical intervention and contributes to improved patient outcomes [12,13,23,26,27].

### 2.4. Surgical Treatment

Surgical excision is the recommended treatment for operable MPTs [11,17,28,29,30,31]. As for other breast tumors, the choice of surgery should take into account both oncological and cosmetic outcomes [2,11,17,29]. If an adequate margin and a good cosmetic outcome can be obtained with a wide excision, breast-conserving surgery should be the first choice of treatment. NCCN guidelines recommend at least a 1 cm excision margin if preoperative assessments suggest the presence of a borderline tumor or MPT, while wide margins are not required for excision of benign PTs [7,18,28,31,32].

However, the evidence of a closer margin at final pathology should be discussed in a multidisciplinary context as according to NCCN guidelines, mastectomy is not an absolute indication in these cases [7,11,33,34,35,36,37,38].

Mastectomy is recommended when the preoperative surgical assessment estimates an inability to obtain adequate margins without causing cosmetic deformities that would be unacceptable to the patient [7,17,19,28,31,39,40]. While tumor size is not associated with LR [12,41], most studies agree that patients with evidence of narrow surgical margins at final pathology have higher LR rates than those with negative margins [11,23,36,37,42,43,44,45]. This encompasses a systematic review and meta-analyses of 9234 individual cases, with 18% of these patients having an MPT; among them, a positive surgical margin was significantly linked to an increased risk of local recurrence (OR 6.85; 95% CI 1.58–29.64) [29,30,31,32]. However, a meta-analysis of four studies including 162 patients demonstrated no differences in LR between ≥1 cm and <1 cm margins [34,46].

In current NCCN guidelines, there are no clinical scenarios in which axillary staging is recommended [7], so there is no indication to perform axillary surgery [7,17,22,28,47,48,49]. A palpable lymphadenopathy is noted in 10–15% of patients presenting with an MPT but is usually reactive to tumor necrosis and infected or ulcerated skin lesions, with <1% of lymph nodes being pathologically involved [50].

### 2.5. Adjuvant Radiotherapy

There is no consensus on whether RT improves overall survival (OS) or disease-free survival (DFS) in patients with an MPT as reported by Chao X et al. in their systematic review and metanalysis [51]. Current guidelines from the NCCN and the European Society for Medical Oncology (ESMO) suggest that radiotherapy may be considered for patients with an MPT that have high-risk features, such as positive surgical margins, tumor size greater than 5 cm, or high-grade pathology [7,52].

A recent update of SEER analysis published in 2024 reported results of a larger cohort of 2261 patients, who were diagnosed and treated in the last two decades. In this larger cohort, at least 20% of patients (20, 12%) underwent radiotherapy. Results showed that in terms of OS and breast cancer-specific survival, RT did not have a significant impact [53].

These findings underscore the role of margin status in the decision to administer RT, suggesting that it may primarily serve as a tool for local control rather than improving long-term survival outcomes. In conclusion, while adjuvant radiotherapy may be beneficial for certain high-risk patients with an MPT—particularly those with positive margins or high-grade histology—the evidence supporting its universal application remains limited. Prospective trials and long-term follow-up studies are needed to refine the guidelines and better delineate which subgroups of patients will benefit most from RT after excision. Until then, treatment decisions should be personalized, carefully considering tumor biology, surgical outcomes, and patient preferences.

### 2.6. Systemic Treatment

The role of chemotherapy for MPTs is controversial and should only be considered after consultation with an expert center [13].

Many investigators have confirmed the low efficacy of monochemotherapy (cyclophosphamide, ifosfamide, or doxorubicin). Currently, treatments typically involve doxorubicin and ifosfamide, and are similar to those for soft tissue sarcoma (STS) as this combination showed the most activity and greatest impact on survival [49]. Neoadjuvant therapy appears to hold a similar significance, yet some recent case reports indicate that aggressive treatment with alkylating agents should be pursued when feasible [54].

Approximately 20% of patients with MPT develop metastases. Generally, research on systemic therapy is limited to small case reports and series that often highlight positive outcomes. However, in practice, patients appear to receive less benefit from chemotherapy, experiencing relatively short durations of response compared to those with more common sarcoma histologies [55].

Given the rarity of the disease, new therapeutic possibilities are yet to be explored in phase II/III trials.

## 3. Primary Breast Sarcoma

Breast sarcomas comprise a heterogeneous group of non-epithelial neoplasms. They are rare tumors, accounting for <1% of breast neoplasms and <5% of soft tissue sarcomas. The annual incidence is approximately 4.5 cases per million [56,57].

The average size at diagnosis is larger compared to epithelial tumors, with a mean diameter of about 3 cm at the time of pathological analysis, although lesions up to 40 cm in diameter have been described [58]. The overlying skin and the nipple-areola complex are rarely infiltrated, except for cases of angiosarcoma where thickening, erythema, and/or red-violet discoloration of the skin overlying the lesion may occur [59].

PBSs, like sarcomas in other body sites, are poorly responsive to chemo- and radiotherapeutic treatments, although the role of the latter is still debated; currently, the main treatment remains radical surgical excision [60,61].

### 3.1. Epidemiology and Risk Factors

PBSs predominantly affect women, with a peak incidence in the fifth to sixth decades of life, although they can occur at any age [60,61,62]. The most common subtype is angiosarcoma, which accounts for approximately 30–50% of breast sarcomas [62,63].

In some patients, genetic factors (e.g., Li–Fraumeni syndrome or neurofibromatosis type 1) and environmental factors (exposure to radiation, vinyl chloride, alkylating agents, and immunosuppressive agents) may be implicated [58], while chronic lymphedema after mastectomy and axillary lymph node dissection is a reported risk factor for the onset of lymphangiosarcoma (Stewart–Treves syndrome) [64].

### 3.2. Pathologic Assessment

PBSs are histologically heterogeneous, and different classifications have been proposed [4]. The classification referred to is currently established by the WHO in 2020 [9].

In a Canadian national case series of 991 patients with a PBS—excluding angiosarcoma—the distribution of histological subtypes was as follows: unspecified sarcomas 26%, spindle cell sarcoma 14%, leiomyosarcoma (LMS) 12%, giant cell sarcoma 10%, stromal sarcoma 6%, malignant fibrous histiocytoma 6%, myxoid fibrosarcoma 4%, dermatofibrosarcoma protuberans 3%, fibrosarcoma 3%, undifferentiated sarcoma 3%, liposarcoma (LPS) 3%, pleomorphic liposarcoma 2%, and others 8% [65].

Grading represents an essential prognostic factor in sarcomas, including those of mammary origin: the elements considered for grading are the extent of tissue differentiation, mitotic count, presence/absence of necrosis, cellularity, and pleomorphism; angiosarcoma is an exception for which the literature results are conflicting [66].

Primary angiosarcoma may develop spontaneously but is more usually seen in patients who received radiotherapy to the affected breast [67]. These lesions show poorly formed irregular vascular channels infiltrating the stroma with pleomorphic hyperchromatic endothelial cells exhibiting high mitotic activity [68,69]. They often have hematoma-like areas due to extensive vascular proliferation and infiltrative growth without well-defined borders. Additional features include the presence of epithelioid cells in some variants and frequent hemorrhage [69,70,71]. Angiosarcomas must be differentiated from benign vascular lesions like hemangiomas, which show well-formed vascular channels lined by regular endothelial cells and lack significant atypia and mitotic activity [69,72]. Tumor cells are positive with a wide range of endothelial markers, such as CD31, CD34, D2-40, and ERG [73]. In post-radiation angiosarcoma, a strong and diffuse nuclear positivity, with a loss or reduced expression of H3K27me3 can be observed [74,75].

The diagnosis of mesenchymal lesions in the breast relies on a combination of histopathological examination, immunohistochemical staining, and molecular studies where necessary [76]. Immunohistochemical markers are critical in differentiating between lesions; for instance, CD34 is useful in distinguishing myofibroblastomas and spindle cell lipomas, while cytokeratins can help identify metaplastic carcinoma in the differential diagnosis of PTs [76,77]. Molecular studies such as fluorescence in situ hybridization FISH or polymerase chain reaction PCR can be employed in challenging cases to detect specific genetic alterations like MDM2 amplification in well-differentiated LPS [78]. Common challenges include histological overlap between benign and malignant lesions, heterogeneity within the same tumor, especially in PTs and LPSs, and ensuring sufficient tissue sampling for accurate diagnosis [76,79]. Understanding these histological features is essential for pathologists and clinicians as accurate histological assessment guides clinical management, ranging from conservative follow-up for benign lesions to aggressive surgical and adjuvant therapy for malignant mesenchymal tumors [80].

### 3.3. Diagnosis

Although studies concerning imaging findings in breast sarcomas are limited due to their low incidence, it seems that they have some characteristic radiological findings [58,81].

Breast sarcomas on mammography often appear as a noncalcified hyperdense mass, with indistinct or circumscribed margins borders [58,81,82]; these findings may resemble benign fibroadenomas [31,83]. The lack of calcifications and the oval shape of these lesions distinguish them from epithelial tumors; however, these same characteristics are also described in PTs [58]. Furthermore, mammography may appear normal in the presence of a breast sarcomatous lesion [83].

Ultrasound sonography is not specific for radiological diagnosis of breast sarcomas. They usually appear as irregular and hyperechoic lesions with no shadowing [81,82,83], although hypoechoic lesions with indistinctive borders and a posterior acoustic shadow have been described [58].

Contrast-enhanced MRI is the preferred imaging technique as it helps detect suspicious malignant changes, such as irregularly shaped oval masses that exhibit T2 hyperintensity along with rapid and uneven contrast enhancement and washout [31,58,82,83]. Additionally, MRI thoroughly evaluates the disease’s local spread, highlighting the extent of involvement in adjacent skin, fascia, and muscle structures, which is vital for effective surgical planning and subsequent radiotherapy [31].

A core biopsy should be performed with needles 16 G or bigger [58], and immunohistochemical analysis of specific cytokeratins should be performed [31,81].

All patients with a diagnosis of breast sarcoma should be assessed for distant disease. It is advisable to perform computed tomography (CT) of the chest, abdomen, and pelvis, along with a bone scan. Lungs are the most common site of metastases globally, while angiosarcoma has a propensity for secondary spread to the liver and bone [31].

The role of positron emission tomography (PET) scans remains unclear in the assessment of breast sarcomas [31,58].

### 3.4. Surgical Treatment

It is strongly recommended that breast sarcomas be managed at a specialized sarcoma referral center to improve overall survival (OS) by investigating clinicopathological features and a multidisciplinary approach [31,81].

There is widespread acceptance that surgical resection should be the first modality of treatment for primitive breast sarcoma [58,60,82]. However, the best surgical treatment is still debated, and should be tailored to the individual patient. The goal of surgery is local control, and total mastectomy has been considered the gold standard for many years [31,58,83]. The choice between mastectomy and breast conservative surgery is based on the possibility to obtain negative margins at final histology [31,58,83,84]: in fact, tumor size and excision margin are the two factors that most impact survival and recurrence [3,37,61,85,86,87]. In a study by Wang et al., the 5-year DFS and OS rates for tumors sized <5 cm, 5–10 cm, and >10 cm, were 75.0% and 88.9%, 70.8% and 75.0%, and 37.5% and 37.5%, respectively [88].

It is also advisable to evaluate the tumor/breast size ratio before surgery to properly perform correct planning of the surgical strategy [60]. While some studies advocate for mastectomy, showing increased OS over conservative surgery, others have demonstrated no significant advantage [3,4,87,88,89,90,91,92].

For smaller localized breast sarcomas, most authors recommend wide excision with a margin greater than 1 cm [82,83,86]. In the case of angiosarcomas, it is recommended to obtain at least a 3 cm clear margin during surgery since these lesions often have an infiltrative cutaneous disease that extends well beyond the visible tumor [82,83,86]. In this set of patients, mastectomy with delayed reconstruction remains the most prudent surgical approach [58,83].

Because the aesthetic outcome of breast-conserving surgery when approaching large resections is often poor, some authors suggest oncoplastic reconstruction with parenchymal rearrangement or reconstruction with a local flap as a viable option [83,93].

Sentinel lymph node biopsy is not routinely performed in patients with breast sarcomas as nodal dissemination is very rare [58,82,83,84]. In a study by Fong et al. involving 1772 cases of STS, the rate of nodal metastasis was only 2.6%, while in a study based on SEER and focused on breast sarcomas, 129 out of 333 patients underwent lymphadenectomy, and only 6 patients (4.7%) were found to have nodal metastases [94,95]. Radical dissection of axillary lymph nodes is appropriate in the presence of histologically proven isolated nodal disease or where the purpose of surgery is local control and symptom relief [58,83].

### 3.5. Adjuvant Radiotherapy

Breast sarcomas are rare malignant tumors for which treatment protocols are not well-established. Adjuvant RT is recommended after a positive margin resection in tumors larger than 5 cm and in any high-grade sarcoma because of the high risk of LR [13,96]. Since PBSs are sometimes associated with p53 mutation and Li–Fraumeni syndrome, testing for these pathogenic variants should occur before administering adjuvant RT [58].

### 3.6. Systemic Treatment

To the best of our knowledge, there are no randomized controlled trials specifically focused on primary breast sarcomas. As previously noted, the available studies—and those discussed in the following sections—address soft tissue sarcomas more broadly. Current guidelines extend the recommendations from these studies to the treatment of breast sarcomas [7,8].

#### 3.6.1. First-Line Treatments

Surgery remains the cornerstone of treatment for primary breast sarcomas (PBSs), although a subset of patients present with locally advanced or metastatic disease at diagnosis, making resection unfeasible [13,97]. In these cases, neoadjuvant chemotherapy may be considered, especially for high-grade tumors or those larger than 5 cm [98]. The standard perioperative regimen combines anthracyclines and ifosfamide, with doxorubicin as the backbone of first-line therapy [7,99,100]. Histology-driven neoadjuvant approaches have not demonstrated superior outcomes in terms of DFS or OS and are not routinely recommended [100]. Whether doxorubicin should be administered as a monotherapy or in combination remains a matter of debate, with recent studies reporting divergent conclusions [101,102] (Table 2). 

The response to chemotherapy remains highly variable and must be tailored to histotype sensitivity in a multidisciplinary setting [55,82]. Among new agents, aldoxorubicin—an albumin-binding prodrug—has shown reduced cardiotoxicity and favorable PFS outcomes in relapsed or refractory STS compared to other treatments including doxorubicin and dacarbazine [103,104,105] (Table 3). Additional options under investigation include amrubicin, a third-generation anthracycline with potentially reduced cardiac toxicity [106], and gemcitabine/docetaxel, which showed no superiority to doxorubicin and higher toxicity in the GeDDiS trial [107,108] (Table 3). 

Weekly paclitaxel has demonstrated sustained activity in angiosarcomas and remains a potential first-line option for this subtype [7,109].

#### 3.6.2. Second-Line Treatments

Second-line options include agents with modest efficacy compared to first-line regimens. Dacarbazine remains one of the oldest available drugs, used as monotherapy or in combination with gemcitabine; the latter is supported by phase II data and NCCN category 2B recommendation [7,110,111]. Eribulin and trabectedin are active mainly in selected subtypes [112,113]. Trabectedin has shown improved PFS over dacarbazine in randomized trials involving patients previously treated with anthracyclines and at least one other systemic regimen [114,115,116]. Temozolomide, a prodrug of dacarbazine, has demonstrated moderate activity in pretreated sarcomas when administered on a prolonged schedule, though phase III data are lacking [117,118].

#### 3.6.3. New Treatment in the Target Therapy Era

To the best of our knowledge, no randomized controlled trials specifically evaluated targeted therapies in primary breast sarcomas. As with other systemic approaches, the available evidence is extrapolated from studies conducted on soft tissue sarcomas, without specifying whether or how many breast sarcomas were included in the analyses.

Among new treatment agents, Tivozanib, another TKI targeting VEGFR and PDGFR, has demonstrated modest PFS in heavily pretreated metastatic STS [119]. Anlotinib, also targeting VEGFR, FGFR, and PDGFR, significantly improved PFS compared to placebo in advanced STS [120]. Regorafenib showed an improvement in PFS for doxorubicin-pretreated nonadipocytic sarcomas [121]. Additionally, several PD-1/L1 inhibitors (e.g., pembrolizumab, nivolumab, camrelizumab, durvalumab, retifanlimab, sintilimab, and toripalimab) are being investigated in combination with chemotherapy, showing promising responses in undifferentiated pleomorphic sarcoma and other subtypes [122,123,124,125]. NTRK inhibitors like larotrectinib and entrectinib have also demonstrated efficacy in NTRK fusion-positive sarcomas, advancing personalized medicine [126].

## 4. Conclusions

Malignant mesenchymal tumors of the breast are rare but extremely aggressive, presenting significant challenges in both diagnosis and treatment. Due to the lack of standardized guidelines, clinical decision-making remains complex and varies widely. Surgical treatment remains the cornerstone of therapy with the primary goal of achieving complete excision to prevent local recurrences and metastases. Surgical options include both breast- conserving surgery and mastectomy, with axillary treatment typically only necessary in cases of biopsy-proven nodal involvement. While the roles of radiotherapy and chemotherapy are still unclear, ongoing research is essential to refine these treatment modalities. The rarity of these tumors complicates the conduction of large-scale studies, making it vital for healthcare professionals to adopt a tailored, multidisciplinary approach, especially in high-volume centers specializing in rare malignancies. These settings provide the best opportunity for improving patient outcomes through expertise and collaboration. Ultimately, advancing our understanding of malignant mesenchymal tumors, enhancing diagnostic precision, and developing comprehensive treatment protocols will be key to improving patient prognoses and managing these challenging malignancies more effectively.

## Figures and Tables

**Table 1:** Current evidence on malignant mesenchymal tumors of the breast - focus on sarcomas and phyllodes tumors.

Study (Year)	Global Cohort	5y-OS	5y-DFS	5y-LRR	Median Follow-Up	Key Findings	Study Limitations
	(Number of Patients)				(Period of Study)		
Holm et al. [5] (2015)	42	49%	48%	24%	50.4 (1979–2014)	- Tumor size and grade are significant prognostic factors	- Retrospective study
							- Single institution
							- Small and disomogeneous cohort
							- Missing data
Confavreux et al. [3] (2006)	70	49%	28%	-	31.7 (1966–2004)	- Negative surgical margins (R0) significantly improve survival (72% vs. 38%)	- Retrospective study
							- Small and disomogeneous cohort
						- Angiosarcomas have the worst DFS (7%)	- Lack of uniform treatment protocols
McGowan et al. [4] (2000)	78	57%	47%	26%	114 (1958–1990)	- Negative surgical margins (R0) improve local relapse-free survival (80% vs. 33%)	- Retrospective study
							- Single institution,
						- Radiation dose >48 Gy improves 5-year OS (91% vs. 50%)	- Small and disomogeneous cohort
							- Missing or incomplete data

OS: Overall Survival; DFS: Disease Free Survival; LRR: Local Recurrence Rate.

**Table 2:** Randomized Controlled Trials on Sarcomas’ First-line treatments.

Study	Patients (n)	Population	Median Follow-Up	Comparative Arms	PFS/OS	Clinical Insights
Judson et al. [101] (EORTC 62012)	455	Advanced, unresectable or metastatic high-grade STS:	56 months	Doxorubicin alone (n = 228)	PFS 4.6 vs. 7.4 months (HR = 0.74; *p* = 0.003)	Combination improves PFS only. Use in selected, fit patients. Higher toxicity
		UPS, myxoid or round cell liposarcoma, pleomorphic and dedifferentiated liposarcoma, pleomorphic rhabdomyosarcoma, synovial sarcoma, myxofibrosarcoma, fibrosarcoma, leiomyosarcoma, angiosarcoma, malignant peripheral nerve sheath tumour, epithelioid sarcoma, unclassified high-grade sarcoma (not otherwise specified)		vs.		
				Doxorubicin and Ifosfamide (n = 227)	OS 12.8 vs. 14.3 months (HR = 0.83; p = 0.076)	
Pautier et al. [102] (LMS-0)	150	Metastatic or relapsed unresectable leiomyosarcomas (uterine and soft tissue leiomyosarcomas) that had not previously been treated with chemotherapy	37 months	Doxorubicin alone (n = 76)	PFS 6.2 vs. 12.2 months (HR = 0.41; *p* < 0.0001)	Strong PFS in LMS. Promising for first-line. Awaiting OS data. *
				vs.		
				Doxorubicin + Trabectedin (n = 74)		

STS: Soft Tissue Sarcomas; UPS: undifferentiated pleomorphic sarcoma; PFS: Progression Free Survival; OS: Overall Survival; LMS: Leiomyosarcomas. * OS data are not yet mature and further analysis is needed.

**Table 3:** Randomized Controlled Trials on new cytotoxic drugs for soft tissue sarcomas.

**ALDOXORUBICIN—Randomized Controlled Trials**						
**Study (Year)**	**Patients (n)**	**Population**	**Endpoints**	**Median Follow-Up**	**Comparative Arms**	**Key Results**
Chawla et al. [103] 2015	123	Locally advanced, unresectable, and/or metastatic high grade STS (Leiomyosarcoma, Liposarcoma, Fibrosarcoma, Synovial sarcoma, Others *)	PFS, OS	13 months	Aldoxorubicin	PFS: 8.3 vs. 4.6 months
					(n = 83)	(*p* < 0.001)
					vs.	
						OS: 15.8 vs. 14.3 months
						(*p* = 0.21; HR = 0.73)
					Doxorubicin	
					(n = 40)	
Chawla et al. [105] 2017	433	Relapsed/refractory STS (Leiomyosarcoma, Liposarcoma, Synovial sarcoma, L-sarcomas subgroup, Others *)	PFS	-	Aldoxorubicin	Overall PFS: 4.06 vs. 2.9 months
						(*p* = 0.12; HR = 0.82)
					vs.	
						L-sarcomas PFS: 5.32 vs. 2.96 months
						(*p* = 0.007; HR = 0.62)
					Investigators’ choice (Dacarbazine, Doxorubicin, Pazopanib, Ifosfamide, Gemcitabine/Docetaxel)	
**GEMCITABINE—DOCETAXEL—Randomized Controlled Trials**						
**Study (Year)**	**Patients (n)**	**Population**	**Endpoints**	**Median Follow-Up**	**Comparative Arms**	**Key Results**
Maki et al. [107] 2007	122	Metastatic STS (Leiomyosarcoma, Non-leiomyosarcoma, Liposarcoma, MFH/UPS, Others *)	PFS, OS	-	Gemcitabine-Docetaxel	PFS: 6.2 vs. 3.0 months
					(n = 73)	OS: 17.9 vs. 11.5 months
					vs.	
					Gemcitabine alone	
					(n = 49)	
GeDDis trial [108] 2017	257	Previously untreated advanced unresectable or metastatic STS (Uterine/Non-uterine leiomyosarcoma, Synovial sarcoma, Pleomorphic sarcoma, Others *)	PFS	22 months	Doxorubicin	PFS: 23.3 vs. 23.7 weeks (*p* = 0.06; HR = 1.28)
					(n = 129)	
						No treatment-related deaths (2 deaths due to disease + treatment)
					vs.	
					Gemcitabine-Docetaxel	
					(n = 128)	

STS: Soft Tissue Sarcomas; MFH: Malignant Fibrous Histiocytoma; UPS: Undifferentiated Pleomorphic Sarcoma; PFS: Progression Free Survival; OS: Overall Survival. * no otherwise specified.

## Data Availability

Not applicable.

## Abbreviations

The following abbreviations are used in this manuscript:

PTs	Phyllodes Tumors
WHO	World Health Organization
MPT	Malignant Phyllodes Tumors
PBS	Primary Breast Sarcoma
RT	Radiation Therapy
HPF	High-power fields
NCCN	National Comprehensive Cancer Network
MRI	Magnetic Resonance Imaging
ADC	Apparent Diffusion Coefficient
LR	Local Recurrence
OS	Overall Survival
DFS	Disease-free survival
ESMO	European Society for Medical Oncology
SEER	Surveillance, Epidemiology, and End Results
BCSS	Breast Cancer-Specific Survival
STS	Soft Tissue Sarcoma
CT	Computed tomography
PET	Positron Emission Tomography
PFS	Progression-Free Survival
ORR	Objective Response Rate
EORTC	European Organisation for Research and Treatment of Cancer
LPS	Liposarcoma
LMS	Leiomyosarcoma
LVEF	Left Ventricular Ejection Fraction
DCR	Disease Control Rate
MLPS	Metastatic Myxoid-Liposarcoma
PLRs	Platelet–lymphocyte Ratios
BSC	Best Supporting Care
TKI	Tyrosine Kinase Inhibitor
CR	Complete Remission
PR	Partial Response
ICI	Immune Checkpoint Inhibitors
UPS	Undifferentiated Pleomorphic Sarcoma
ASPS	Alveolar Soft Part Sarcoma
O	Olaratumab
G	Gemcitabine
D	Docetaxel
